# Immune-Related Adverse Events in PD-1 Treated Melanoma and Impact Upon Anti-Tumor Efficacy: A Real World Analysis

**DOI:** 10.3389/fonc.2021.749064

**Published:** 2021-11-26

**Authors:** Melissa L. Bastacky, Hong Wang, Dylan Fortman, Zahra Rahman, Gerard P. Mascara, Timothy Brenner, Yana G. Najjar, Jason J. Luke, John M. Kirkwood, Hassane M. Zarour, Diwakar Davar

**Affiliations:** ^1^ Department of Pharmacy, University of Pittsburgh Medical Center, Pittsburgh, PA, United States; ^2^ Graduate School of Public Health, University of Pittsburgh, Pittsburgh, PA, United States; ^3^ Department of Medicine, University of Pittsburgh Medical Center, Pittsburgh, PA, United States; ^4^ University of Pittsburgh Medical Center (UPMC) Hillman Cancer Center, Pittsburgh, PA, United States

**Keywords:** melanoma, metastatic, immunotherapy, CTLA-4, PD-1, immune related adverse events, irAE, autoimmune

## Abstract

**Background:**

Anti-PD-1 immune checkpoint inhibitor (ICI) therapy has revolutionized the treatment of melanoma by producing durable long-term responses in a subset of patients. ICI-treated patients develop unique toxicities - immune related adverse events (irAEs) – that arise from unrestrained immune activation. The link between irAE development and clinical outcome in melanoma and other cancers is inconsistent; and little data exists on the occurrence of multiple irAEs. We sought to characterize development of single and multiple irAEs, and association of irAE(s) development with clinical variables and impact upon outcomes in advanced melanoma patients treated with anti-PD-1 ICIs.

**Methods:**

We conducted a retrospective study of 190 patients with metastatic melanoma treated with single-agent anti-PD-1 ICI therapy between June 2014 and August 2020 at a large integrated network cancer center identified through retrospective review of pharmacy records. irAEs were graded based on the Common Terminology Criteria for Adverse Events (CTCAE) version 5.0.

**Results:**

190 patients were evaluated of whom 114 patients (60.0%) experienced ≥1 irAE, including 30 (15.8%) with grade 3/4 irAEs. The occurrence of any irAE was strongly associated with the development of investigator-assessed response to anti-PD-1 therapy (p < 0.0001); whether evaluated by current (p=0.0082) or best (p=0.0001) response. In patients with ≥2 irAEs, distinct patterns were observed. Median progression-free survival (PFS) and overall survival (OS) were greater in those with any irAE compared to those without (PFS, 28 months *vs*. 5 months, p < 0.0001; OS, not reached *vs*. 9 months, p < 0.0001). Development of ≥2 irAEs had a trend towards improved PFS and OS compared to those who developed a single irAE, although this did not reach statistical significance (p=0.2555, PFS; p=0.0583, OS). Obesity but not age or gender was distinctly associated with irAE development.

**Conclusions:**

In this study, we demonstrated that irAE occurrence was significantly associated with response to anti-PD-1 therapy and improved PFS/OS. Those who developed multiple irAEs had a trend towards improved PFS and OS compared to those who developed only a single irAE. Increased BMI but neither age nor gender were associated with irAE development. Distinct patterns of irAEs observed suggest shared etiopathogenetic mechanisms.

## Introduction

Programmed cell death protein 1 (PD-1) is a receptor expressed on activated T cells which binds to PD ligand 1 (PD-L1, B7-H1) and PD ligand 2 (PD-L2, B7-DC) (1–4). The interaction between PD-1 and PD-L1 negatively regulates T cell function (1–4). Within tumors, tumor cells and tumor-infiltrating immune cells including macrophages and dendritic cells (DCs) express PD-L1 (5); and the PD-1/PD-L1 interaction down-regulates host immune responses in peripheral tissues, representing a means by which tumors subvert anti-tumor immune responses. PD-1/PD-L1 blockade increases the number and function of tumor antigen (TA)-specific CD8+ T cells preclinically and in *ex vivo* melanoma (6, 7). Therapeutic blockade of the PD-1/PD-L1 interaction by immune checkpoint inhibitors (ICI) has transformed the management of advanced melanoma; with objective responses in 35-40% of patients (8, 9), with 35% progression-free at 1-year (10, 11), and 34% alive at 5-years (12).

Immune-related adverse events (irAE) represent a unique spectrum of toxicities observed in patients treated with ICIs targeting cytotoxic T-lymphocyte–associated protein 4 (CTLA-4) and PD-1 receptors. irAEs can affect any organ system with unique patterns and incidence depending on whether patients are treated with single agent or dual ICI. irAEs most commonly involve skin, endocrine glands, gastrointestinal tract and liver (13, 14). irAE occurrence has been associated with improved outcomes for patients treated with anti-PD-(L)1 or anti-CTLA-4 ICI primarily in the context of melanoma (15–19), non-small cell lung cancer (NSCLC) (13, 20, 21), and urothelial cancer (19). Separately, while certain demographic variables including body mass index (BMI) (22, 23), and gender (24), have been associated with ICI outcomes; the association of these with irAE occurrence have not been studied.

In this study, we report upon the association with irAE occurrence and outcome to PD-1 blockade in patients with advanced melanoma treated with nivolumab or pembrolizumab. We evaluate the temporal pattern of irAE occurrence, relationship of irAE occurrence in relation to outcomes including objective response, progression-free and overall survival, and relationship of irAE occurrence. We perform separate analyses by gender and BMI and investigate the influence of systemic steroid use upon the stated outcomes.

## Materials And Methods

### Patient Selection

Approval was obtained from the University of Pittsburgh Cancer Institute (UPCI) Institutional Review Board (IRB) for a retrospective analysis of cancer patients who had received treatment with anti-PD-1 and/or anti-CTLA-4 ICI (IRB number PRO18080253) identified based on retrospective review of pharmacy records. For the purposes of this analysis, only patients with advanced metastatic melanoma treated with single-agent anti-PD-1 ICI were considered. Inclusion criteria were diagnosis of unresectable (stage III) or metastatic (stage IV) cutaneous, mucosal, or unknown primary melanoma, treatment with at least one dose of anti-PD-1 therapy administered singly, and at least one restaging scan available to assess disease response. Patients with uveal melanoma were excluded. A prespecified sample size was not determined. 190 melanoma patients treated between June 2014 and August 2020 with either nivolumab or pembrolizumab in the advanced setting were included, although patients who received adjuvant nivolumab or pembrolizumab were not included. Patients were treated with intravenous pembrolizumab (2mg/kg every 21 days; 200mg every 21 days) or nivolumab (3mg/kg every 14 days; 240mg every 14 days; 480mg every 28 days) under a range of doses and schedules as indicated. Treatment duration was not specified *a priori* but was typically administered for up to 24 months, disease progression, or unacceptable toxicity.

Patient demographics and baseline characteristics, along with data regarding prior systemic therapy (if any) and performance status (PS) on the Eastern Cooperative Oncology Group (ECOG) scale were collected. Mutational status as determined on primary or metastatic tumor tissue was recorded. Disease stage at start of anti-PD-1 therapy was assigned using the American Joint Committee on Cancer (AJCC) 8th edition staging classification (25).

Radiographic response was assessed using computed tomography (CT) scans or positron emission tomography (PET) scans with CT attenuation performed at baseline and every 3 months as assessed by the treating investigator (D.D., Y.N., J.J.L., or J.M.K.). Clinical response to treatment was categorized as complete response (CR), partial response (PR), stable disease (SD), or progressive disease (PD) according to the Response Evaluation Criteria in Solid Tumors (RECIST) v 1.1 criteria (26). Patients who were not radiologically evaluable were not included in this analysis. Best overall response was defined as the best achieved response observed during treatment period; while current objective response rate (ORR) was defined as the proportion of patients with CR or PR at the most recent radiographic assessment. Progression-free survival (PFS) was defined as time from date on which immunotherapy was started to date of first radiographic or clinical progression. Patients who had not progressed were censored at the date of last follow up. Overall survival (OS) was calculated from date on which immunotherapy was started to date of death; and patients who were alive were censored at the date of last follow up.

### Immune-Related Adverse Event (irAE) Assessment

irAE terms were defined as any AE that previously had been reported to be associated with the mechanism of action of pembrolizumab or nivolumab ICI therapy. The following groups of irAEs were considered based on a prespecified list and were grouped as follows (1): endocrine irAE (hypothyroidism, hyperthyroidism, hypophysitis, type 1 diabetes, or adrenal insufficiency) (2); cutaneous irAE (macular rash, papular rash, bullous disease, vitiligo excluding pruritus alone) (3); rheumatologic irAE (myalgia, arthritis, myositis, temporal arteritis, xerostomia) (4); gastrointestinal irAE (colitis, hepatitis, pancreatitis) (5); other irAEs (pneumonitis, immune thrombocytopenia, nephritis, aseptic meningitis). We specifically only evaluated irAEs that occurred after receipt of anti-PD-1 therapy and any event temporally linked to this; but specifically excluded any irAE that occurred following receipt of any subsequent therapy (immune, targeted therapy or other). We excluded AEs – such as fatigue or pruritus – that are not classically considered immune mediated but could conceivably result from an immune mediated process. irAEs that occurred between the start and last dose of treatment were tabulated. irAEs severity was graded according to the National Cancer Institute’s Common Terminology Criteria for Adverse Events, version 5.0. Management of irAEs was obtained based on chart review. Treatment-related irAEs were typically managed following published American Society of Clinical Oncology (ASCO) clinical practice guidelines and use of steroid and steroid-sparing agents was documented (27, 28).

To describe patterns of multiple irAEs, we evaluated all patients with >1 irAE (N=44) that developed during treatment and/or follow-up period. irAE categories were grouped and between-category relationships (rheumatologic-endocrine, cutaneous-rheumatologic, etc.) were considered regardless of number, and/or temporality and subsequently enumerated (_x_C_y_, where x is the number of irAE categories, and y is the number of unique rearrangements). This generated 3 relationships per individual from those with 3 irAEs (N=10), and 6 relationships per individual from those with 4 irAEs (N=4). The total number of unique two-dimensional relationships so derived (78) were plotted on a chord gram using Flourish studio (https://app.flourish.studio/@daan/chord-diagram).

### Statistical Analysis

Data were summarized using descriptive statistics. Normality testing (D’Agostino and Pearson test) was performed to determine whether data were sampled from a Gaussian distribution. The Mann–Whitney U test was used to compare continuous non-normally distributed variables. Incidence of events among the groups was analyzed for statistical significance using the Fisher exact test. Logistic regression models were used to study the effect of explanatory variables on binary outcomes, and odds ratios (OR) and 95% confidence intervals (CI) were calculated based on these models. Survival outcomes were evaluated with the Kaplan–Meier method. The relationship between survival outcomes with risk factors was studied with Cox models. Hazard ratio (HR) and 95% CI were calculated for each risk factor. Univariate and multivariate analysis were performed when appropriate, using Cox proportional hazard model. All p values were two-sided; p values < 0.05 were statistically significant. Statistical analysis was performed with SAS version 9.4 (SAS institute Inc, Cary, NC), GraphPad Prism version 6.0 for Mac (GraphPad Software, San Diego CA), and IBM-Microsoft SPSS (SPSS Statistics. International Business Machines Corporation IBM. 2013. Armonk, USA) version 20.0 for Mac.

## Results

### Patients and Clinical Characteristics

190 patients with advanced/unresectable cutaneous, mucosal, or unknown primary melanoma who met the inclusion criteria were included in the present analysis (119 males, 71 females). The median age was 68 (range 20.0 to 91.0 years), and the median duration of follow-up for all patients was 18.0 months. 162 (85.3%) of patients had stage IV disease while 28 (14.7%) had unresectable stage III disease. 157 (82.6%) of patients were treated with pembrolizumab; while 33 (17.4%) received nivolumab. Anti-PD-1 therapy was typically administered in the front-line setting (107 patients, 56.3%), and less commonly in the 2^nd^ (47 patients, 24.7%) or ≥3^rd^ line (36 patients, 19.0%) settings. 68 (35.8%) of patients were exposed to ipilimumab previously in either the adjuvant or metastatic settings. The overall rate of progression was 59.5%, and patients who progressed were treated with either targeted therapy, or investigational immunotherapy. Baseline characteristics are tabulated in **Table 1**.

**Table 1:** Baseline characteristics of PD-1 treated advanced melanoma patients.

**Sex**	
• Male	119 (62.6%)
**Age**	
• Median (range)	68 (20–91)
• ≤60	63 (33.2%)
• 60 to ≤70	55 (28.9%)
• >70	72 (37.9%)
**BMI**	
• <25	53 (27.9%)
• 25-30	65 (34.2%)
• >30.0	72 (37.9%)
**Histology**	
• Cutaneous	145 (76.3%)
• Unknown primary	30 (15.8%)
• Mucosal	15 (7.9%)
**ECOG PS**	
• 0-1	163 (85.8%)
• 2	11 (5.8%)
• ≥3	2 (1.0%)
• NR	14 (7.4%)
**Prior autoimmune disease**	23 (16.0%)
**Extent of disease**	
• Unresectable stage III	28 (14.7%)
• M1a	26 (13.7%)
• M1b	28 (14.7%)
• M1c	59 (31.1%)
• M1d	49 (25.8%)
**Mutational status**	
• ^#^BRAF V600E/K	52 (27.4%)
• ^$^NRAS	43 (22.6%)
**LDH (ULN = 171)**	
• Normal	41 (21.6%)
• 1 to ≤2x ULN	99 (52.1%)
• >2x ULN	28 (14.7%)
• N/A	22 (11.6%)
**Anti PD-1 Agent Received**	
• Nivolumab	33 (17.4%)
• Pembrolizumab	157 (82.6%)
**Anti-PD-1 Line of Therapy (Advanced Setting)**	
• 1^st^	107 (56.3%)
• 2^nd^	47 (24.7%)
• ≤3^rd^	36 (19.0%)
**Prior Therapy**	
• Anti-CTLA	68 (35.8%)
• Interferon	59 (31.0%)
**Best Response to Anti-PD-1**	
• CR	36 (19.0%)
• PR	55 (28.9%)
• SD	25 (13.1%)
• PD	74 (39.0%)
**Current Response to Anti-PD-1**	
• CR	30 (15.8%)
• PR	37 (19.5%)
• SD	10 (5.2%)
• PD	113 (59.5%)

^#^BRAF mutation status was unknown in 14 (7.3%) of patients.

^$^NRAS mutation status was unknown in 32 (16.9%) of patients.

### IrAE Incidence, Temporal Relationship and Resolution

**Figure 1:**
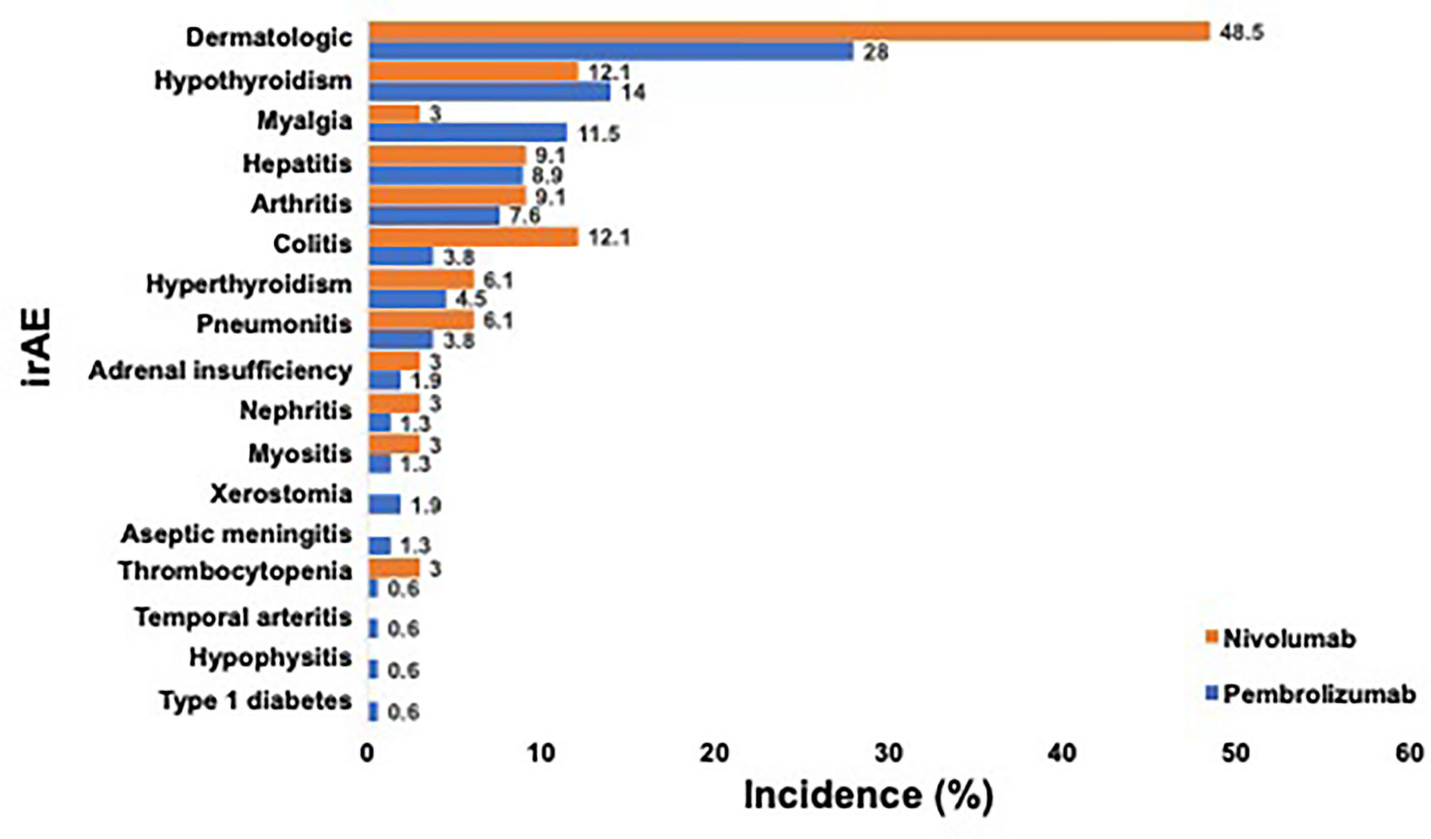
Incidence of Any-grade irAE Occurring in at least One PD-1 Treated Advanced Melanoma Patient. a. Legend: Incidence of any-grade irAE that occurred in ≥1 patient after anti-PD-1 therapy for advanced melanoma is shown. Patients who received pembrolizumab (blue) and nivolumab (orange) are depicted separately. Incidence of each irAE is listed on the right.

Treatment-related irAEs of any grade occurred in 114 (60.0%) of patients, and 44 (23.2%) of patients experienced more than one AE (**Figure 1**). The vast majority of all irAEs were Grade 1-2 and comprised: dermatologic irAEs [61/68 (89.7%)], endocrinologic irAEs [39/41, (95.1%)], rheumatologic irAEs [34/41, (82.9%)]. Grade 3-4 irAEs occurred in 30 (15.8%) patients; while no incidence of grade 5 irAEs were noted. Anti-PD-1 therapy resulted in treatment discontinuation in 30 (15.8%) of patients, with the most common etiology being pneumonitis in 6 patients (20.0%), colitis in 5 patients (16.7%), and hepatitis in 5 patients (16.7%).

**Figure 2:**
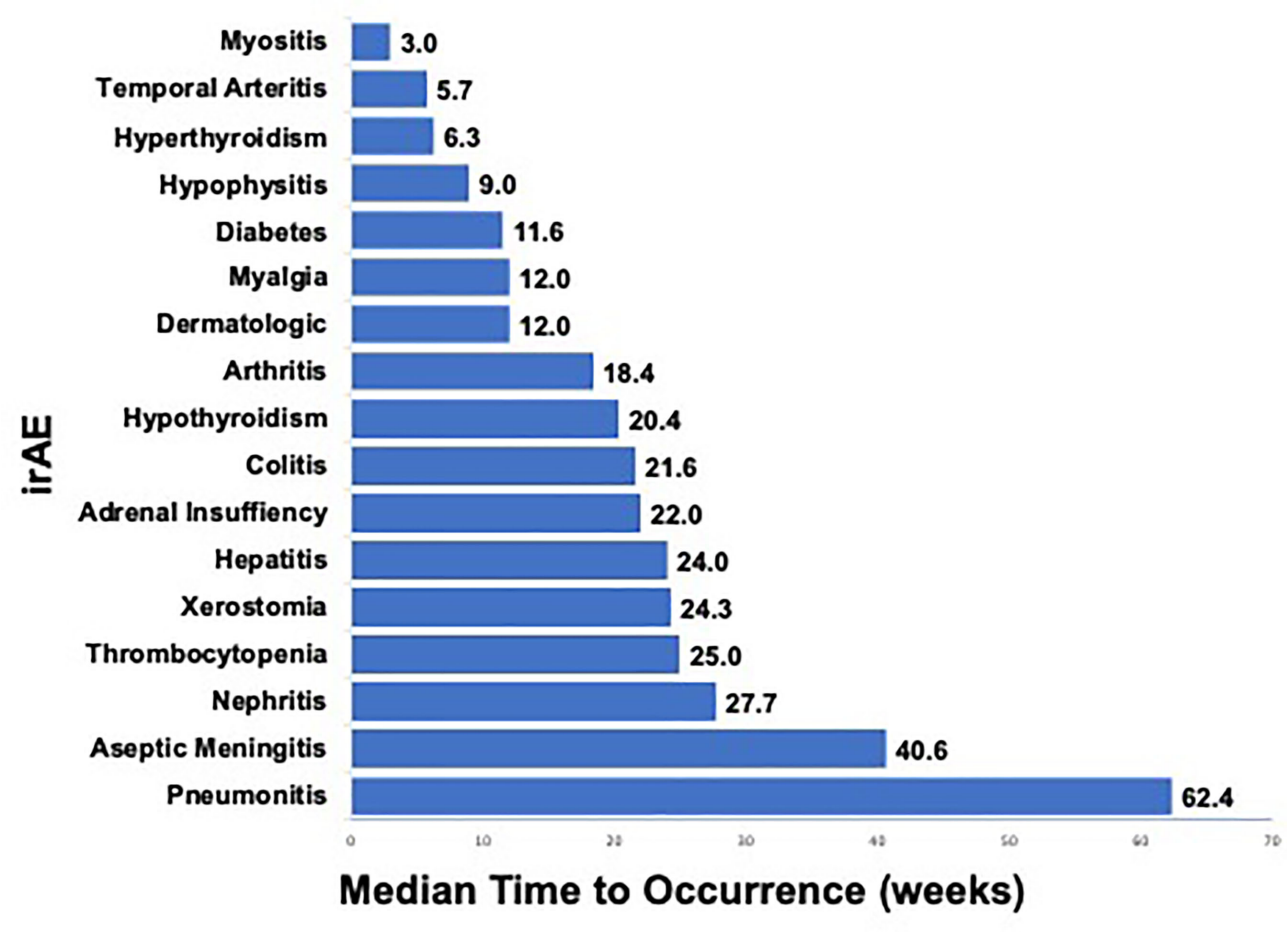
Median Time (weeks) to irAE Occurrence in PD-1 Treated Advanced Melanoma Patients. a. Legend: Median time (in weeks) from start of therapy to development of any-grade irAE that occurred in ≥1 patient after anti-PD-1 therapy for advanced melanoma is shown. Range (in weeks) from start of therapy to development of irAE is shown on right.

When we evaluated the various irAEs in relation to start of therapy, we observed differing times to occurrence among the various categories of irAEs (**Figure 2**). Dermatologic irAEs tended to occur relatively soon after initiation of therapy (median 12.0 weeks) while neurologic and pulmonary irAEs tended to occur relatively late (median 40.6 weeks and 62.4 weeks respectively) (**Figure 2**). The median time (in weeks) to onset of the most common irAEs (incidence ≥5%) were as follows: hyperthyroidism (6.3 weeks), dermatologic (12.0 weeks), myalgia (12.0 weeks), arthritis (18.4 weeks), hypothyroidism (20.4 weeks), colitis (21.6 weeks), hepatitis (24.0 weeks), and pneumonitis (62.4 weeks) (**Figure 2**); qualitatively similar to prior reports (14). At the time of this analysis, all irAEs had completely resolved except for vitiligo, myalgias and endocrine irAEs requiring permanent hormone replacement therapy.

### IrAE Occurrence and Response/Survival to Anti-PD-1 Therapy

**Figure 3:**
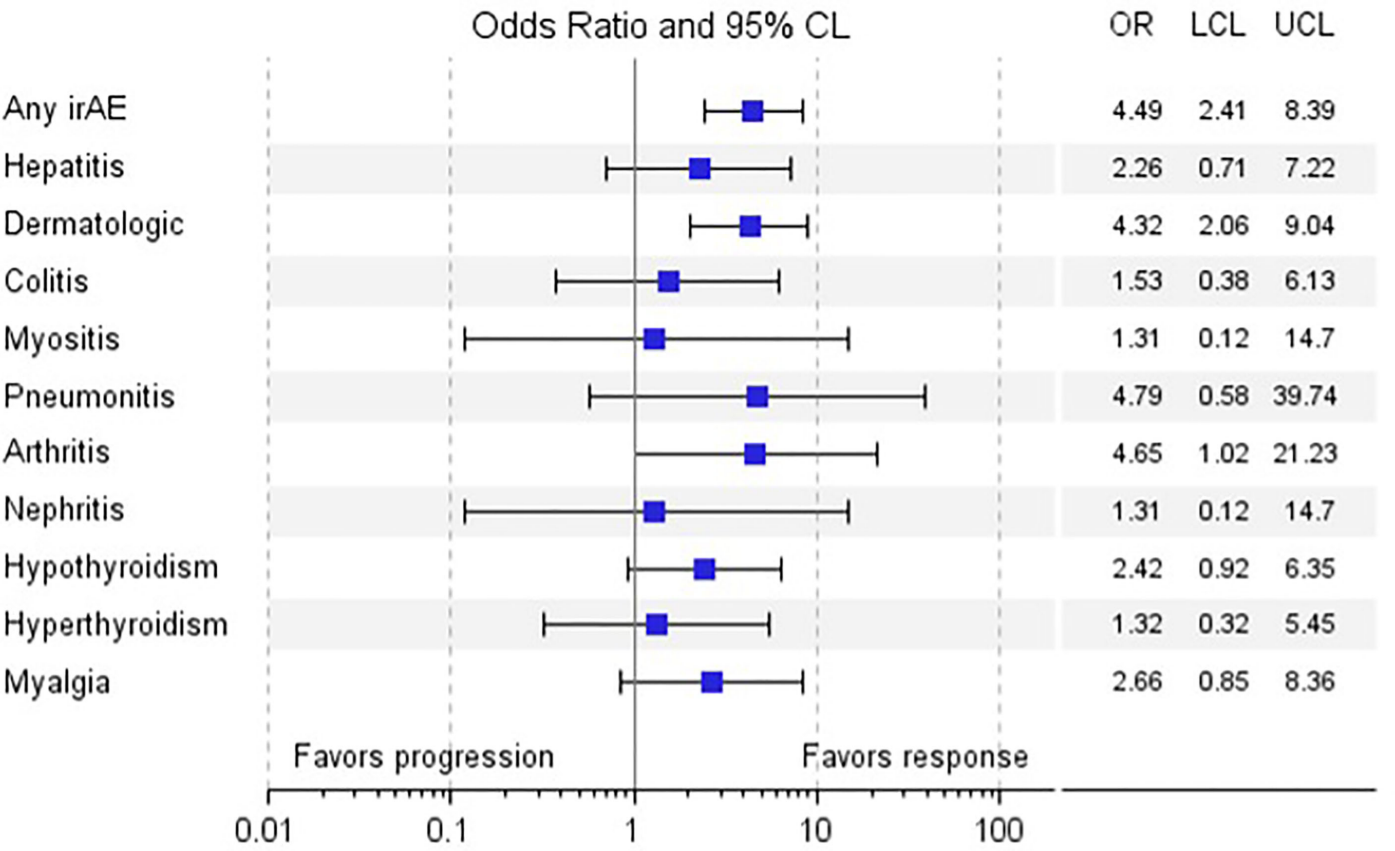
Odds Ratio of Response *vs*. Non-response by irAE. a. Legend: Forest plot of odds ratio of response *vs*. non-response by various irAE.

The occurrence of any irAE was strongly associated with the development of investigator-assessed response to anti-PD-1 therapy, defined as CR or PR assessed by RECIST v1.1 (p<0.0001); whether evaluated by current (p=0.0082) or best (p=0.0001) response, summarized in **Table 2** and **Supplementary Table 1**. Of the various irAEs, only dermatologic and rheumatologic irAEs were significantly associated with current response (dermatologic, p=0.0006; rheumatologic, p=0.0197), and best response (dermatologic, p<0.0001; rheumatologic, p=0.0157). The odds of response in patients who developed any irAE, a dermatologic irAE, or arthritis irAE were 4.49 (95% CI 2.41, 8.39), 4.32 (95% CI 2.06, 9.04), and 4.65 (95% CI 1.02, 21.23), respectively, summarized in **Table 3** and **Figure 3**. These results underscore similar observations by other groups in the setting of melanoma patients treated with anti-PD-1 ICIs both in the adjuvant and advanced settings (16, 19, 29–32).

Summary of irAE occurrence by response (Current).

**Table 2A:** 

irAE	N (%) in Each Group			p-value with Fisher’s exact test (comparing CR/PR *vs*. SD/PD^#^)
	CR/PR	SD	PD	
**Any irAE**	87	21	84	0.0082
**Dermatologic**				
• Rash	26 (29.9%)	4 (19.0%)	20 (23.8%)	0.0006
• Vitiligo	9 (10.3%)	1 (4.8%)	5 (6.0%)	
• Bullous dermatoses	1 (1.1%)	0 (0%)	1 (1.2%)	
• Dry skin	0 (0%)	0 (0%)	1 (1.2%)	
**Endocrine**				
• Hypothyroidism	9 (10.3%)	4 (19.0%)	13 (15.5%)	0.8440
• Hyperthyroidism	1 (1.1%)	2 (9.5%)	6 (7.1%)	
• Hypophysitis	1 (1.1%)	0 (0%)	0 (0%)	
• Adrenal insufficiency	1 (1.1%)	2 (9.5%)	1 (1.2%)	
• Type I DM	0 (0%)	1 (4.8%)	0 (0%)	
**Rheumatologic**				
• Myalgia	12 (13.8%)	0 (0%)	7 (8.3%)	0.0197
• Xerostomia	2 (2.3%)	0 (0%)	1 (1.2%)	
• Temporal arteritis	0 (0%)	0 (0%)	1 (1.2%)	
• Myositis	1 (1.1%)	0 (0%)	2 (2.4%)	
• Arthritis	9 (10.3%)	1 (4.8%)	5 (6.0%)	
**Gastrointestinal**				
• Colitis	5 (5.7%)	0 (0%)	5 (6.0%)	0.1888
• Hepatitis	1 (1.1%)	5 (23.8%)	11 (13.1%)	
• Pancreatitis	0 (0%)	0 (0%)	0 (0%)	
**Pneumonitis**	5 (5.7%)	0 (0%)	3 (3.6%)	0.1327
**Nephritis**	1 (1.1%)	1 (4.8%)	1 (1.2%)	1.0000
**Neurologic**				
• Aseptic meningitis	1 (1.1%)	0 (0%)	1 (1.2%)	1.0000
**Hematologic**				
• Hemolytic anemia	0 (0%)	0 (0%)	0 (0%)	0.1231
• Immune thrombocytopenia	2 (2.3%)	0 (0%)	0 (0%)	

^#^Based on investigator-assessed best response to anti-PD-1 therapy based on RECIST v1.1.

**Table 2B:** Summary of irAE occurrence by response (Best).

irAE	N (%) in Each Group			p-value with Fisher’s exact test (comparing CR/PR *vs*. SD/PD^#^)
	CR/PR	SD	PD	
**Any irAE**	121	35	36	0.0001
**Dermatologic**				<0.0001
• Rash	35 (28.9%)	6 (17.1%)	9 (25%)	
• Vitiligo	12 (9.9%)	1 (2.9%)	2 (5.6%)	
• Bullous dermatoses	1 (0.8%)	0 (0%)	1 (2.8%)	
• Dry skin	0 (0%)	1 (2.9%)	0 (0%)	
**Endocrine**				0.4465
• Hypothyroidism	15 (12.4%)	5 (14.3%)	6 (16.7%)	
• Hyperthyroidism	3 (2.5%)	3 (8.6%)	3 (8.3%)	
• Hypophysitis	1 (0.8%)	0 (0%)	0 (0%)	
• Adrenal insufficiency	1 (0.8%)	3 (8.6%)	0 (0%)	
• Type I DM	1 (0.8%)	0 (0%)	0 (0%)	
**Rheumatologic**				0.0157
• Myalgia	15 (12.4%)	0 (0%)	4 (11.1%)	
• Xerostomia	2 (1.6%)	1 (2.9%)	0 (0%)	
• Temporal arteritis	1 (0.8%)	0 (0%)	0 (0%)	
• Myositis	1 (0.8%)	1 (2.9%)	1 (2.8%)	
• Arthritis	10 (8.3%)	4 (11.4%)	1 (2.8%)	
**Gastrointestinal**				0.8365
• Colitis	6 (5.0%)	1 (2.9%)	3 (8.3%)	
• Hepatitis	6 (5.0%)	7 (20.0%)	4 (11.1%)	
• Pancreatitis	0 (0%)	0 (0%)	0 (0%)	
**Pneumonitis**	5 (5.0%)	2 (5.7%)	1 (2.8%)	0.4832
**Nephritis**	2 (1.6%)	0 (0%)	1 (2.8%)	0.6077
**Neurologic**				0.2281
• Aseptic meningitis	2 (1.6%)	0 (0%)	0 (0%)	
**Hematologic**				0.2281
• Hemolytic anemia	0 (0%)	0 (0%)	0 (0%)	
• Immune thrombocytopenia	2 (1.6%)	0 (0%)	0 (0%)	

^#^Based on investigator-assessed best response to anti-PD-1 therapy based on RECIST v1.1.

**Table 3:** Univariate logistic regression analysis for the association of irAE with any response.

irAE	Parameter estimate	Standard error	p-value	Odds ratio (95% CI)
Any irAE	1.50	0.32	<.0001	4.49 (2.41, 8.39)
Hepatitis	0.82	0.59	0.1681	2.26 (0.71, 7.22)
Dermatologic	1.46	0.38	0.0002	4.32 (2.06, 9.04)
Colitis	0.43	0.71	0.5450	1.53 (0.38, 6.13)
Myositis	0.27	1.23	0.8269	1.31 (0.12, 14.70)
Pneumonitis	1.57	1.08	0.1465	4.79 (0.58, 39.74)
Arthritis	1.54	0.77	0.0473	4.65 (1.02, 21.23)
Nephritis	0.27	1.23	0.8269	1.31 (0.12, 14.70)
Hypothyroidism	0.88	0.49	0.0721	2.42 (0.92, 6.35)
Hyperthyroidism	0.28	0.72	0.7002	1.32 (0.32, 5.45)
Myalgia	0.98	0.58	0.0934	2.66 (0.85, 8.36)

**Figure 4:**
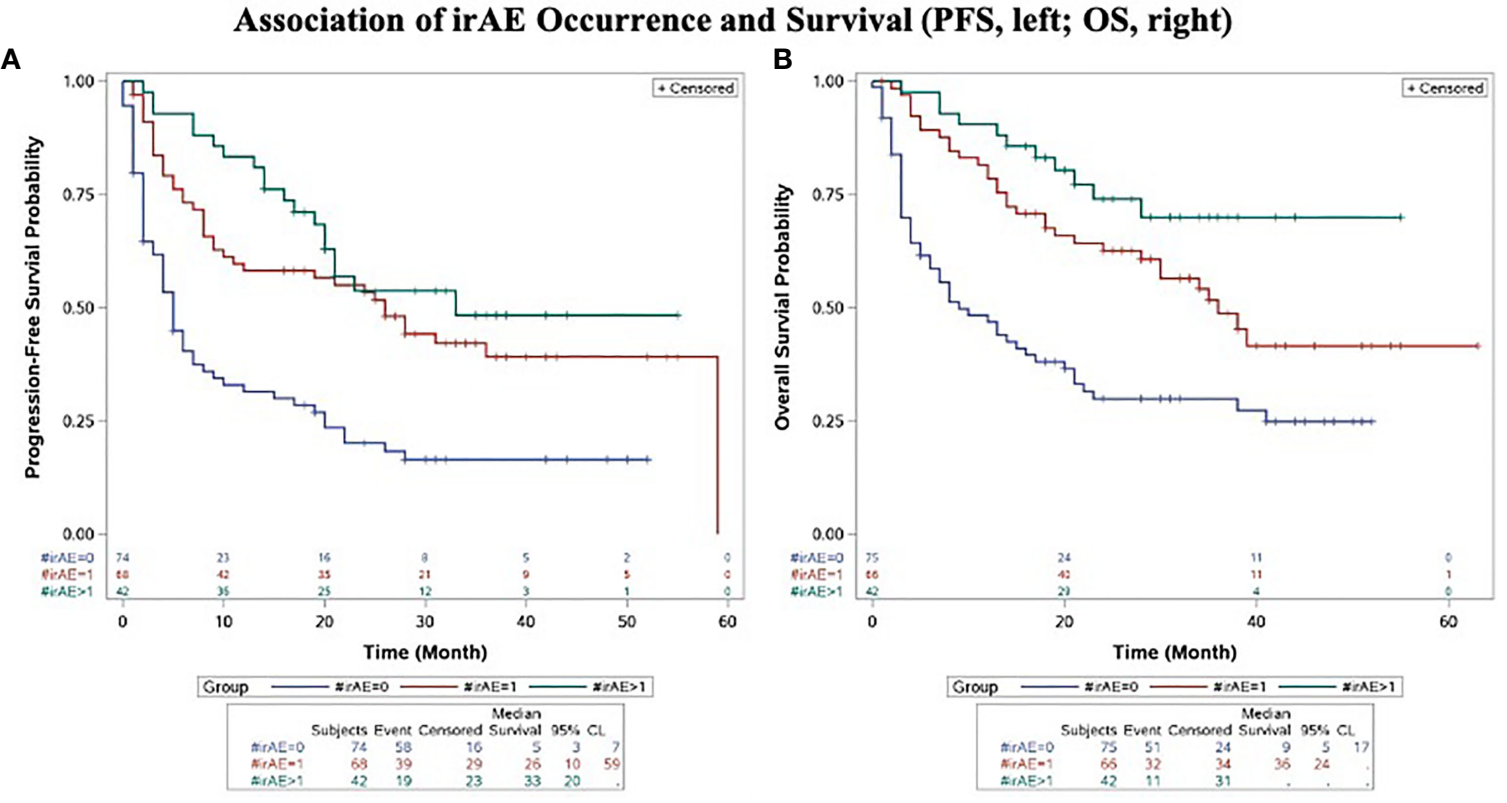
Association of irAE Occurrence and Progression-Free Survival **(A)** and Overall Survival **(B)**. Kaplan-Meier plots of progression-free survival **(A)** and overall survival **(B)** in advanced melanoma patients treated with anti-PD-1 therapy compared by development and extent of irAE (0 *vs*. single *vs*. multiple). All p-values significant and unadjusted for multiple comparisons.

Concordant with the positive association of irAE and objective response to anti-PD-1 therapy, irAE occurrence was associated with improved PFS and OS. Median PFS (mPFS) and OS (mOS) were significantly greater in those who developed irAE than in those who did not (mPFS 28 months *vs*. 5 months, p < 0.0001; mOS not reached *vs*. 9 months, p < 0.0001) (**Figures 4A, B**).

The proportion of patients who were BRAF mutant was 27.4%, while 22.6% of patients were NRAS mutant. Oncogenic *BRAF* mutations were associated with lower odds of ORR (odds ratio 0.37, p=0.0049) and irAE development (odds ratio 0.45, p=0.0180). Conversely, we observed greater ORR (odds ratio 2.53, p=0.0177) and irAE development (odds ratio 1.49, p=0.2844) in *NRAS* mutant patients; concordant with prior observations by other groups (33). Given that this was a retrospective study wherein mutation status was not prospectively collected with consequent imbalances, the associations of oncogenic drivers with irAE occurrence must be interpreted cautiously.

The longitudinal nature of the analysis permitted us to characterize patients who developed multiple irAEs. Overall, 42 (22.1%) unique patients developed 100 unique irAEs, grouped by organ system involvement in **Supplementary Table 2**. Three patients experienced four distinct irAEs; while 10 distinct patients developed three distinct irAEs (**Supplementary Table 2**). The commonest irAE doublets were dermatitis-endocrinopathy (15, 19.5%) and dermatitis-rheumatologic (15, 19.5%), followed by endocrinopathy-hepatitis (7, 9.1%) and endocrinopathy-rheumatologic (6, 7.8%) – a pattern reflected partially by the overall incidence of organ-specific irAEs. These relationships are depicted using a chord diagram in **Supplementary Figure 1**.

Patients who developed multiple irAEs had improved PFS and OS compared to those who did not develop an irAE (p<0.0001, PFS; p<0.0001, OS). Compared to those who developed a single irAE, patients who developed multiple irAEs had a trend towards improved PFS and OS, although this did not reach statistical significance for the OS and the PFS comparisons (p=0.2555, PFS; p=0.0583, OS) (**Figures 4A, B**).

### Relationship of Steroid Use Upon irAE Resolution and Relationship of Gender, Age and BMI on irAE Development

50 (26.3%) patients required steroids for treatment of irAEs, of whom 7 (14.0%) patients failed steroid management and required biologics. Measures of response to therapy including objective response rate (ORR), median progression-free survival (PFS) and median overall survival (OS) were similar among patients who received steroids for irAE management compared to those who did not: ORR 47.1% *vs*. 52.9% (p = 0.2825); mPFS 31 months *vs*. 26 months (p = 0.5186); and mOS not reached *vs*. 38 months (p = 0.2593) (**Supplementary Table 3**).

We investigated the influence of gender, age at treatment onset and pre-treatment BMI upon irAE development. While irAE occurrence was not affected by sex (p=0.9026) or age (p=0.2130), we observed that increasing BMI (OR 1.06, p = 0.0206) increased the likelihood of irAE occurrence (**Supplementary Table 4**). Herein, we observed that the odds of irAE development were greater in BMI >25.0 (OR 1.97, 95% CI 1.04, 3.74) compared to patients with BMI ≤25.0. These data are in line with other reports including a meta-analysis linking increased BMI with a greater risk of irAE development in patients treated with anti-PD-1 and/or anti-CTLA-4 ICI (34–38).

## Discussion

Several single-center and pooled analyses have evaluated the incidence of irAE and outcomes to anti-PD-1 therapy in melanoma in multiple settings including adjuvant therapy and advanced disease (15–19, 39). However, this real-world single-center analysis is the first study to our knowledge that evaluates the longitudinal development of irAE in anti-PD-1 treated melanoma patients and their relationship to response and survival outcomes along with other demographic determinants of response and autoimmunity including age, gender and BMI. Given the inherent difficulty in classifying and attributing irAEs to antecedent anti-PD-1 therapy, it is not surprising that the data on the incidence and severity of immune-related toxicities is disparate.

Our data show that most irAEs from anti-PD-1 monotherapy were low-grade. The incidence, time to occurrence and profile of irAEs and grade 3/4 irAEs were similar to what had been previously reported for pembrolizumab and nivolumab in the setting of advanced melanoma (8, 9, 39). The majority of irAEs we observed resolved without sequelae, except for endocrine irAEs such as adrenal insufficiency and hypothyroidism, which often required long-term endocrine therapy. The longitudinal follow up at a single center afforded us the ability to examine the temporal trends of irAEs along with the co-occurrence of multiple irAEs. While dermatologic and endocrine irAEs were common events that occurred early, we did note moderately frequent co-occurrence of dermatologic-rheumatologic and dermatologic-endocrine manifestations - suggestive of the presence of a shared factor (or factors) at the level of the host shared among these patients such as: HLA polymorphisms (40), tumor antigenic mimicry (20), and/or intestinal microbiota (41–43). Prospective studies integrating phenotypic information with other biomarkers are needed to further characterize these patterns.

The association between irAE occurrence and improved benefit to anti-PD-1 ICI in terms of response and PFS has previously been reported in ICI-treated melanoma patients (14, 17); although a recent pooled analysis of KEYNOTE-001, KEYNOTE-002, and KEYNOTE-006 studies that corrected for immortal time bias suggested similar ORR, PFS and OS statistics among patients who did or did not experience irAEs (39). While we observed that irAE occurrence was associated with response to anti-PD-1 ICI, this association was driven overwhelmingly by the positive association between radiographic response and irAEs belonging to either dermatologic or rheumatologic categories. The positive association between cutaneous adverse events (particularly vitiligo) and favorable therapeutic outcomes in melanoma has been shown with various agents including biochemotherapy (44, 45), interferon (46), ipilimumab (47), and anti-PD-1 ICI (48, 49). Given the known associations between cutaneous and autoimmune diseases with joint/muscle involvement, these observations suggest a shared etiopathogenetic mechanism between cutaneous and rheumatologic irAEs wherein exposure to anti-PD-1 ICI unmasks a latent reactivity to shared antigen(s) (50).

Female gender, increasing age and greater BMI are all associated with increased risk of autoimmunity and autoimmune diseases (51–53). In the context of gender, this is likely related to the effects of endogenous estrogen and/or prolactin upon self-reactive B and T cells (54–56), and/or skewed X-chromosome inactivation (XCI) that results in loss of immunological tolerance to self-antigens (57). While several reports have suggested improved immunotherapy efficacy in men (24), other meta-analyses have derived opposite conclusions (58, 59). We did not observe any differences in irAE incidence by sex.

Both ageing and obesity are tightly associated with “inflammaging” – a chronic low-grade inflammatory state characterized by inflammatory cytokine production and a dynamic reorganization of the adaptive immune compartment associated with co-emergence of exhausted T cells expressing inhibitory receptors including PD-1 and CD4^+^CD25^high^FoxP3^+^ regulatory T cells (60, 61). Consequently, both increased age (62–64), and elevated BMI (65, 66), have independently been linked with improved outcomes to ICI. Consistent with a prior report, we did not observe an increase in the risk of irAE with increased age (62). We observed a significant association between irAEs and higher BMI – consistent with results from retrospective studies evaluating anti-PD-1 ICI-treated cancer patients (34, 38), along with a recent meta-analysis and systemic review (36).

This study has several limitations. Similar to other retrospective analyses, this study is susceptible to inherent biases including reporter bias, although detailed chart review by a trained oncology pharmacist was utilized to minimize this. As we sought to characterize irAEs in patients treated with anti-PD-1 ICI singly, we restricted our analyses to melanoma patients which limits the generalizability of these results to other histologies and patients treated with combinations including ICI/chemotherapy and ICI combinations. Although our focus was development of irAEs following anti-PD-1 monotherapy, we did consider the impact of prior anti-CTLA-4 therapy on clinical outcomes. However, upon review of the data, we identified significant heterogeneity in CTLA-4 treated patients (i.e. ipilimumab dose, number of cycles given, timing of ipilimumab in respect to subsequent anti-PD-1 therapy, and line of therapy). The retrospective nature of this analyses implied a lack of balance in the number of patients in each CTLA-4 subgroup, precluding statistical analyses. However, given the significant role of CTLA-4 blockade in the melanoma armamentarium hitherto limited by irAEs, and the observations that the irAE incidence of CTLA-4 may be ameliorated by low-dose combinations, future efforts should be directed at evaluating the detailed incidence of irAEs in CTLA-4 treated patients by dose (67). To increase statistical power, we grouped individual irAEs into linked irAE categories. While this approach has been adopted by others, there is the chance that in doing so, irAEs with diametric effects upon response were grouped, negating the overall effect of the irAE category upon response, a particular consideration with endocrinopathies.

In conclusion, the detailed longitudinal analyses in this single-center study permitted deep phenotypic characterization and uncovered previously unreported relationships of multiple temporally separated irAEs; and their relationship to response and survival outcomes in anti-PD-1 treated melanoma patients. These observations suggest that the incidence of certain irAEs may be underreported and that irAEs linked to shared etiopathogenetic mechanisms may co-occur in the same individual weeks to months apart that should be the focus of future analyses.

## Data Availability Statement

The original contributions presented in the study are included in the article/**Supplementary Material**. Further inquiries can be directed to the corresponding author.

## Ethics Statement

The studies involving human participants were reviewed and approved by University of Pittsburgh Cancer Institute (UPCI) Institutional Review Board (IRB) (IRB number PRO18080253). Written informed consent for participation was not required for this study in accordance with the national legislation and the institutional requirements.

## Author Contributions

MB, DF, ZR, and HW acquired, analyzed and interpreted data, and helped to draft the manuscript. DD conceived of the study; participated in study design and coordination; acquired, analyzed and interpreted data; and helped to draft the manuscript. All authors contributed to the article and approved the submitted version.

## Funding

This work was supported by Melanoma Research Foundation Breakthrough Consortium (MRFBC) (DD); Award Number P50 CA254865-01A1 from the National Cancer Institute (JK, HZ, DD, and JL); Award Number R01 CA257265-01 from the National Cancer Institute (DD, HZ).

## Conflict of Interest

YN: Research Funding (Merck, Pfizer, and Bristol-Myers Squibb). JL: Stock and Other Ownership Interests (RefleXion); Consulting/Advisory Role (7 Hills, Abbvie, Alnylam, Actym, Alphamab Oncology, Arch Oncology, Array, Bayer, Bristol-Myers Squibb, Checkmate Pharmaceuticals, Cstone, Eisai, EMD Serono, Flame,Fstar, Gilead, Kadmon, KSQ, Knaph, Janssen, Immunocore, Inzen, Macrogenics,Mavu, Merck, Mersana, Nektar, Novartis, Onc.AI, Pfizer, Pyxis, Regeneron, Ribon, Rubius, Silicon, Synlogic, TRex, Tempest, Werewolf, Xilio, Xencor); Research Funding (AbbVie, Agios, Array, Astellas, Bristol-Myers Squibb, Corvus, EMD Serono, Genmab, Ikena, Immatics, Incyte, Kadmon, KAHR, MAcrogenics, Merck, Moderna, Nektar, Numab, Replimmune, Rubius, Spring Bank, Synlogic, Takeda, Trishula, Tizona, Xencor). JK: Consulting/Advisory Role (Amgen, Bristol-Myers Squibb, Checkmate Pharmaceuticals, and Novartis); Research Funding (Amgen, Bristol-Myers Squibb, Castle Biosciences, Checkmate Pharmaceuticals, Immunocore LLC, Iovance, and Novartis). HZ: Consulting/Advisory Role (Bristol Myers Squibb, Checkmate Pharmaceuticals, GlaxoSmithKline/Tesaro and Vedanta Biosciences); Research Funding (Bristol Myers Squibb, Checkmate Pharmaceuticals, and GlaxoSmithKline/Tesaro). DD: Consulting/Advisory Role (Ascendis Pharma, Bristol Myers Squibb, Checkmate Pharmaceuticals, Shionogi, Vedanta Biosciences); Research Funding (Arcus, Checkmate Pharmaceuticals, Cellsight Technologies, GlaxoSmithKline/Tesaro, Merck Inc.).

The remaining authors declare that the research was conducted in the absence of any commercial or financial relationships that could be construed as a potential conflict of interest.

## Publisher’s Note

All claims expressed in this article are solely those of the authors and do not necessarily represent those of their affiliated organizations, or those of the publisher, the editors and the reviewers. Any product that may be evaluated in this article, or claim that may be made by its manufacturer, is not guaranteed or endorsed by the publisher.

## Abbreviations

AJCC, American Joint Committee on Cancer; CR, complete response; CI, confidence intervals; CTLA-4, cytotoxic T-lymphocyte antigen 4; ICI, immune checkpoint inhibitor; IRB, Institutional Review Board; LDH, lactate dehydrogenase; OS, overall survival; PD-1, programmed death 1; PFS, progression-free survival; PR, partial response; RECIST, Response Evaluation Criteria in Solid Tumors; ULN, upper limit normal; HCC, Hillman Cancer Center.
