# Supplementary material for: Immune-Related Adverse Events in PD-1 Treated Melanoma and Impact Upon Anti-Tumor Efficacy: A Real World Analysis
Source: Front Oncol. 2021 Nov 26;11:749064. doi: 10.3389/fonc.2021.749064 (PMC8662734; doi:10.3389/fonc.2021.749064)
Supplement: Supplementary file 1 [file DataSheet_1.docx]

**Supplementary Figure 1: Chord Diagram of irAE Doublets in Patients who Developed ≥2 irAEs**

**
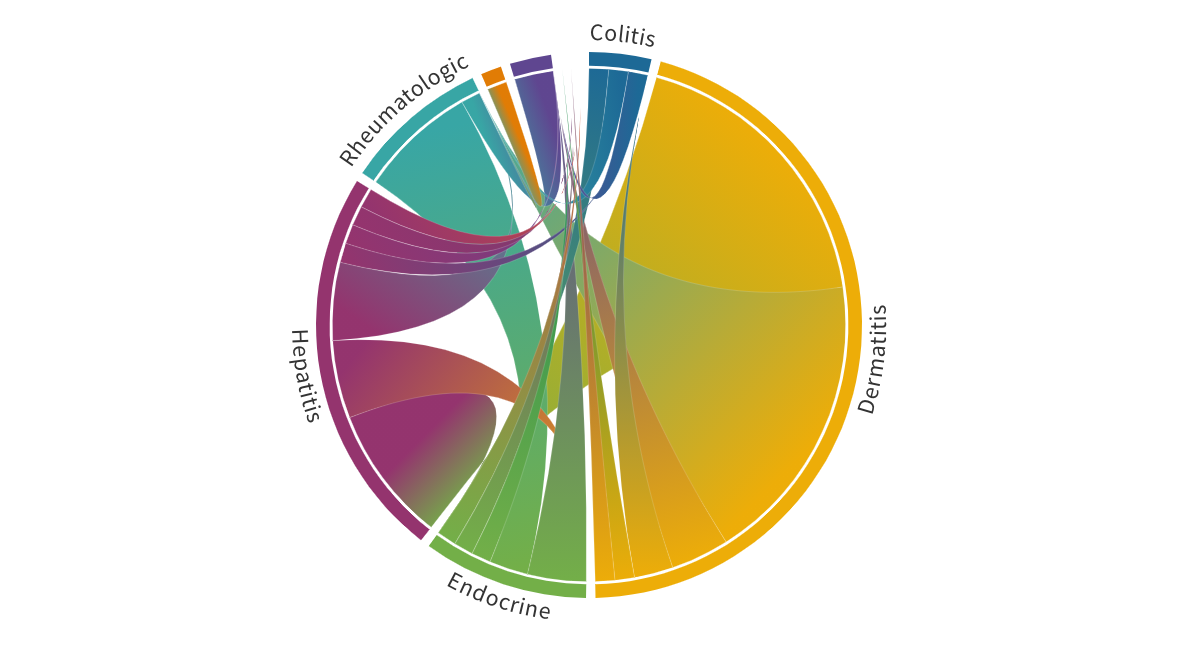
**

**Supplementary Table 1: Summary of Patients with Any irAE Occurrence by Response Status (Any Response, Best Response and Current Response)**

| **Any irAE** | **Number and percent in each responding group** | | **p-value with Fisher’s exact test** |
| --- | --- | --- | --- |
|  | **Any response** | |  |
|  | **R** | **NR** |  |
| No | 30 (26.1%) | 46 (61.3%) | <0.0001 |
| Yes | 85 (73.9%) | 29 (38.7%) |  |
|  | **Best Response** | |  |
|  | **R** | **NR** | 0.0001 |
| No | 23 (25.3%) | 53 (53.5%) |  |
| Yes | 68 (74.7%) | 46 (46.5%) |  |
|  | **Current Response** | |  |
|  | **R** | **NR** | 0.0082 |
| No | 18 (26.9%) | 58 (47.2%) |  |
| Yes | 49 (73.1%) | 65 (52.8%) |  |

**Supplementary Table 2: Summary of Patients who Developed Multiple irAEs**

| **irAE 1** | **irAE 2** | **irAE 3** | **irAE 4** |
| --- | --- | --- | --- |
| Rheumatologic | Endocrinopathy |  |  |
| Dermatitis | Rheumatologic | Endocrinopathy |  |
| Dermatitis | Rheumatologic |  |  |
| Dermatitis | Endocrinopathy |  |  |
| Dermatitis | Endocrinopathy |  |  |
| Dermatitis | Rheumatologic | Pneumonitis |  |
| Dermatitis | Rheumatologic |  |  |
| Dermatitis | Pneumonitis | Rheumatologic |  |
| Dermatitis | Colitis | Rheumatologic |  |
| Dermatitis | Pneumonitis |  |  |
| Dermatitis | Rheumatologic |  |  |
| Dermatitis | Aseptic Meningitis | Endocrine |  |
| Dermatitis | Rheumatologic |  |  |
| Dermatitis | Rheumatologic |  |  |
| Dermatitis | Endocrinopathy |  |  |
| Dermatitis | Endocrinopathy |  |  |
| Dermatitis | Rheumatologic |  |  |
| Dermatitis | Endocrine | Rheumatologic |  |
| Dermatitis | Rheumatologic |  |  |
| Dermatitis | Colitis |  |  |
| Dermatitis | Rheumatologic |  |  |
| Dermatitis | Endocrine |  |  |
| Dermatitis | Endocrine |  |  |
| Dermatitis | Rheumatologic | Endocrine |  |
| Dermatitis | Endocrine |  |  |
| Dermatitis | Endocrine |  |  |
| Hepatitis | Dermatitis |  |  |
| Hepatitis | Dermatitis | Endocrine |  |
| Hepatitis | Rheumatologic |  |  |
| Hepatitis | Rheumatologic |  |  |
| Hepatitis | Colitis | Pneumonitis | Endocrine |
| Hepatitis | Rheumatologic |  |  |
| Hepatitis | Dermatitis | Rheumatologic | Endocrine |
| Hepatitis | Dermatitis | Endocrine | Thrombocytopenia |
| Hepatitis | Endocrine |  |  |
| Hepatitis | Nephritis | Endocrine |  |
| Hepatitis | Endocrine |  |  |
| Endocrine | Rheumatologic |  |  |
| Rheumatologic | Endocrine |  |  |
| Renal | Rheumatologic |  |  |
| Pneumonitis | Endocrine |  |  |
| Pneumonitis | Endocrine | Rheumatologic |  |

**Supplementary Table 3: Summary of Patients with Steroid Use by Response Status in Patients with at Least One irAE. (Any Response, Best Response and Current Response)**

| **Steroid Use** | **Number and percent in each responding group** | | **p-value with Fisher’s exact test** |
| --- | --- | --- | --- |
|  | **Any response** | |  |
|  | **R** | **NR** |  |
| No | 45 (52.9%) | 19 (65.5%) | 0.2825 |
| Yes | 40 (47.1%) | 10 (34.5%) |  |
|  | **Best Response** | |  |
|  | **R** | **NR** | 0.8479 |
| No | 39 (57.4%) | 25 (54.3%) |  |
| Yes | 29 (42.6%) | 21 (45.7%) |  |
|  | **Current Response** | |  |
|  | **R** | **NR** | 1.0000 |
| No | 28 (57.1%) | 36 (55.4%) |  |
| Yes | 21 (42.9%) | 29 (44.6%) |  |

**Supplementary Table 4: Association of BMI, Age, Gender and Mutation Status with irAE Development**

| **Variable** | **Parameter Estimate** | **Standard Error** | **Odds Ratio (95% CI)** | **p value** |
| --- | --- | --- | --- | --- |
| BMI (as a continuous variable)   - >25.0 vs. ≤25.0 - >28.0 vs. ≤28.0 - >30.0 vs. ≤30.0 | 0.054  0.68  0.30  0.36 | 0.023  0.33  0.30  0.31 | 1.06 (1.01, 1.10)  1.97 (1.04, 3.74)  1.35 (0.75, 2.42)  1.43 (0.78, 2.63) | 0.0206  0.0370  0.3135  0.2469 |
| Age (as a continuous variable)   - >50 vs. ≤50 - >60 vs. ≤60 - >70 vs. ≤70 - >80 vs. ≤80 | 0.013  0.40  0.18  0.26  0.25 | 0.011  0.41  0.31  0.31  0.47 | 1.01 (0.99, 1.04)  1.49 (0.67, 3.30)  1.19 (0.65, 2.21)  1.30 (0.71, 2.38)  1.29 (0.52, 3.21) | 0.2130  0.3250  0.5714  0.3932  0.5866 |
| Gender (female vs. male) | 0.019 | 0.15 | 1.04 (0.57, 1.89) | 0.9026 |
| Mutations Status (mutant vs. wild type)   - BRAF V600E/K mutation - NRAS mutation | -0.40  0.20 | 0.17  0.19 | 0.45 (0.24, 0.87)  1.49 (0.72, 3.08) | 0.0180  0.2844 |
